# Improving clinical outcomes of Barrett’s esophagus with high dose proton pump inhibitors and cryoablation

**DOI:** 10.1080/07853890.2023.2191002

**Published:** 2023-05-26

**Authors:** Harry Snady

**Affiliations:** Department of Internal Medicine, RWJBarnabas Health Jersey City Medical Center, Jersey City, NJ, USA

**Keywords:** Barrett’s esophagus, cost-effectiveness, cryoablation, efficacy, endoscopy-interventional, high dose proton pump inhibitors, safety

## Abstract

**Introduction:**

Esophageal adenocarcinoma incidence has increased significantly despite surveillance endoscopy for Barrett’s esophagus (BE) and gastric acid supression medications. This prospective, cohort study’s aims were to determine the long-term efficacy of proton-pump inhibitors twice daily (PPI-BID) with cryotherapy (CRYO) for complete ablation of BE.

**Materials and Methods:**

Consecutive BE patients were managed with a PPI-BID, CRYO ablation, follow-up protocol. Primary outcomes were to determine complete ablation rate of intestinal metaplasia (IM) or dysplasia/carcinoma, and factors affecting recurrence.

**Results:**

Sixty-two patients were enrolled: advanced disease (11%), low-grade or indefinite dysplasia (26%), non-dysplastic BE (63%). In 58 completing CRYO, eradication was confirmed in 100% on surveillance endoscopy. Adverse events (5%) were minor (mild pain 4%). IM recurred in 9% after a mean of 52 months, all successfully re-ablated. No second recurrence occurred. The primary predictor of recurrence was noncompliance with PPI-BID. BE or cardia IM recurred in 35% of those taking proton pump inhibitors once daily or less compared with 0% in those on PPI-BID or dexlansoprazole daily (*p*<.001).

**Conclusions:**

Minimizing acid reflux with at least PPI-BID combined with CRYO ablation appears to be the optimal cost-effective and safe BE treatment for any stage to minimize progression to adenocarcinoma by addressing both the stimulus that causes BE and the presence of goblet cells.

## Key Messages

- Endoscopic surveillance of Barrett’s esophagus (BE) has not made any clear impact on incidence of or mortality from esophageal adenocarcinoma.
- After cost-effective, safe cryoablation of BE, continued effective acid-reflux control with high-dose proton pump inhibitors is critical to minimize recurrence or progression.
- Risk factors and costs will define methods and frequency of limited surveillance after ablation of BE.

## Introduction

Esophageal adenocarcinoma (EAC) can develop in Barrett’s esophagus (BE) with or without intestinal metaplasia (IM). The accepted risk [1–8] is from 200–500/100,000 (0.2 to 0.5%) per year (yr). Since 1975 the incidence of EAC has increased by a factor of >5 despite surveillance endoscopy for BE and suppression of gastric acid with H_2_ blockers and proton pump inhibitors (PPI). Once BE develops there is an 11-fold increase in the risk of EAC [1,6].

For patients with no significant medical problems, the presence of even only nondysplastic (ND) BE/IM causes EAC to become their main health risk. For comparison, the chance of developing any type of cancer between 40 to 60 years old (yo) is about 350/100,000/yr. The chance of developing significant cardiac disease is about 400/100,000/yr. Once dysplasia develops in BE, 2,000–4,000/100,000/yr progress to cancer. If BE of any stage could be eradicated to eliminate almost all risk of EAC without adverse effects, why would a patient not proceed with treatment especially if surveillance could then be minimized as well?

Recent reports of possible serious PPI adverse effects have heightened general concern; although these adverse effects are unconfirmed, avoiding overuse is valid [1,2,9–11]. However, once BE is found, benefits far outweigh risks since PPIs reduce the chance of progression of IM to EAC, although they rarely cause BE to regress or be eliminated [1,2,10,12–14]. Radiofrequency ablation (RFA) has been evaluated extensively to treat the various stages of BE successfully [1,3–6,15–18]. Results for argon plasma coagulation (APC) have been more variable, but a recent study achieved over 90% complete remission over 108 months [19]. However, both methods of ablation cause significant pain and other adverse events such as perforation or stricture [3–6,8,12,17–20]. Studies have shown cryoablation using −196 °C liquid nitrogen (CRYO) to be effective to treat BE even after other of ablation methods including RFA have failed [20,21]. Because (a) benefits/risks ablation of NDBE are still debated despite studies showing its effectiveness [8,22], (b) in 2008 CRYO appeared to have an improved safety profile, and (c) even after a 2015 international consensus conference, there is a lack of consensus of management of BE [1], a prospective, observational long-term follow-up cohort study was designed in 2008 to evaluate the safety and long-term effectiveness of CRYO with proton-pump inhibitors given twice daily (PPI-BID) for treating BE of any stage.

## Methods

### Outcomes

The primary outcomes to be assessed were the rates of (1) complete eradication (CE) of any stage of IM, (2) recurrence, and (3) success of repeat CE. Secondary outcomes were safety, tolerance, adverse events, and analysis of variables associated with recurrence. CE was defined as no IM or dysplasia on histology from treated areas or the proximal cardia. Recurrent BE was defined as any esophageal IM, but not including IM in the cardia (confirmed by presence of oxcyntic cells) or stomach.

### Inclusion and exclusion criteria

Consecutive adult patients with at least BE with IM (goblet cells) on biopsies were eligible for CRYO in a tertiary care outpatient setting. All patients signed informed consent for management (including benefits and risks of treatments versus standard follow-up, and the required long term follow-up) of their extent and grade of BE using Prague circumferential:maximal (C:M) criteria and four quadrant biopsies every 1–2 cm [1,4,7]. Any dysplasia was confirmed by at least two gastrointestinal pathologists including expert second opinion, and at least two separate endoscopies for high grade dysplasia (HGD). Patients with a prior history of EAC treated with endoscopic mucosal resection, surgery or chemoradiotherapy were included.

Patients were excluded if previously treated with RFA. Patients with non-dysplastic BE were excluded if they were not healthy, had significant medical diseases and a life expectancy less than 10 years (www.myabaris.com or American Society Anesthesiology (ASA) II).

Institutional review boards approved all phases of the study protocol (NCT 00526786) with subsequent updated protocols based on interim analyses. Patients were enrolled from 01/2008 through 01/2011 and, after interim analysis, from 08/2014 through 09/2017. Data were collected prospectively on patients followed through March 2022.

### Treatment protocols

**Figure 1.**
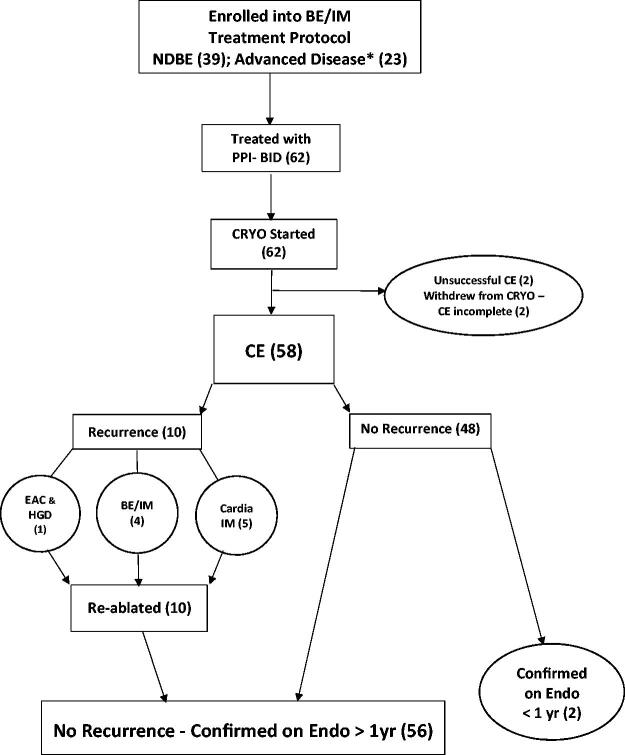
(number): number of patients in category; * EAC (4): while on PPI-BID 3 nodular initially ablated with EMR and 1 flat, epithelial initially ablated with CRYO; BE/IM: Barrett’s esophageal intestinal metaplasia. CE: complete eradication/ablation/response; CRYO: truFreeze cryoablation; EAC: esophageal adenocarcinoma; EMR: endoscopic mucosal resection; Endo: endoscopy; HGD: high grade dysplasia; IM: intestinal metaplasia; NDBE: nondysplastic Barrett’s esophagus; PPI-BID: proton-pump inhibitors given twice daily; yr: year.

Patients were started on PPI such as omeprazole 20 mg twice daily at least two weeks prior to initiation of any endoscopic treatment (Figure 1). Localized carcinoma was treated with either CRYO or, if nodules were present, standard endoscopic mucosal resection prior to CRYO; the site was allowed to heal over six weeks on PPI-BID.

CRYO was then performed with the −196 °C liquid nitrogen truFreeze CryoSpray Ablation System (CSA Medical, Inc., Lexington, MA). Endoscopic sessions were scheduled every three to six weeks to allow for healing as per CSA Medical, Inc. protocols with pre- and post-procedure 10-day clinical follow-up until IM was eliminated/ablated. PPI, such as omeprazole 20 mg, was continued BID until IM was eliminated. In addition, for 10 days following each CRYO session, sucralfate suspension 1 gm TID and famotidine 20 mg at bedtime were added to PPI-BID to maximize healing and minimize the risk of stenosis. This regimen was based on a study from 1989 that showed a similar regimen to be effective in minimizing inflammation/fibrosis after esophageal variceal sclerotherapy [23].

Patients were sedated with propofol and kept in a 30° reverse Trendelenburg position. A dual lumen, modified orogastric suction tube into the stomach kept the intestines decompressed with a nurse monitoring for any abdominal distension. Liquid nitrogen was applied endoscopically through a specialized catheter until the salmon-colored Barrett’s columnar mucosa developed a bright, garnet red color, the objective of CRYO. Regarding dosimetry, CSA Medical, Inc., initially recommended four cycles of 10 s doses for 40 s per site, which was later changed to two cycles of 20 s doses for 40 s per site to try to decrease procedure time. There are currently no comparative dosimetry studies. After various comparisons and adjustments, the current dosimetry is to usually apply three cycles of 15 s for a total of 40–45 s per site; up to six sites/session was usually tolerated.

Aspirin and anti-platelet medications were usually held one day before and after a CRYO session. In one patient, required coumadin was held, and heparin given two days before and after the procedure. Since no bleeding occurred at three CRYO sessions, coumadin was not held for the 4th session; no bleeding occurred even with a therapeutic protime. There are currently no comparative studies.

Follow-up endoscopy was scheduled in three to six months and every one to three years depending on initial pathology. Signs of gastroparesis, defined as the lack of gastric peristalsis and significant retained food at endoscopy after nothing per os >12 h, were always evaluated.

Patients continued PPI-BID for at least one year, and indefinitely if dysplasia had been found initially. For patients with only NDBE (IM) initially, PPI-BID was tapered after one year to once daily, usually dexlansoprazole 60 mg if possible. To assess symptoms, medication regimens, compliance, adverse events, side effects, requirements for surgery, scheduling of endoscopy, and review of the importance of reflux control with PPI-BID for successful outcomes, patients were scheduled for follow-up usually in person or if appropriate by telephone at least every three months until CE. Then, for the next 3 years after CE, follow-up was scheduled depending on clinical course every 3–6 months if they had initial dysplasia or significant symptoms, or every 6–12 months if they had initial NDBE or were asymptomatic. Compliance status with PPI regimen was defined as: (1) compliant – BID-PPI or dexlansoprazole daily taken at least 90% of time; (2) daily compliant – PPI taken at least 90% of time; (3) partially compliant – < 4 PPI doses/week (PRN) for ≤6 months; and (4) noncompliant – no or PRN PPI for > 6 months.

Statistical univariate analysis was performed to assess any association between categorical variables and treatment response with Fisher’s exact T test. Multivariate analysis with logistic regression model was then performed for significant variables. A sample size of 41 was calculated to detect a difference of 80% for progression of BE disease for standard statistical power with a significance level of 0.05. Mean results are reported with ± standard deviation (SD).

## Results

Sixty-two consecutive patients were enrolled; none were lost to clinical follow-up over 80 ± 30mo from initial CRYO. Table 1 shows patient characteristics. In the 58 patients who completed CRYO all sessions, CE was confirmed in 100% on at least one surveillance endoscopy after at least 1 year in 56, and in 2–8 mo in 2 patients (Table 2). Four of 62 patients stopped CRYO prior to eradication due to health or insurance issues. Therefore, on an intention-to-treat-analysis, CE was achieved in 94% (58/62). Non-dysplastic BE was ablated in 38/39 patients (one stopping CRYO after only 1 session). CE required only 1 CRYO session in 19/38 (50%).

**Table 1.** Patient characteristics.

Patient characteristics	Number (%)
Patients	62
Age (years [SD])	62.2 [11.1]
Male : female	32 (52%) : 30(48%)
Race – Caucasian : Other	39(63%) : 23(37%)
Positive human immunodeficiency virus	1 (2%)
C 1.6 cm (range 0–12)	
Barrett’s length (Prague criteria)	M 3.0 cm (range 1–16)
Nondysplastic BE (NDBE)	39 (63%)
Dysplasia	23 (37%)
HGD : LGD or IDD	7 : 16
Intramucosal EAC	4
No aspirin/anti-platelet agent/anticoagulants^a^	49 (79%)
Hiatal hernia	
>5–7cm	2 (3%)
>4–5 cm 7 (11%)	
3–4 cm	8 (13%)
1–2 cm	40 (64%)
None	5 (8%)
Prior gastric obesity surgery	3 (5%)

*Note:* Prague criteria: C: circumferential extent; M: maximum extent in cm; BE: Barrett’s esophagus; HGD: high grade dysplasia; LGD: low grade dysplasia; IDD: indefinite dysplasia; EAC: esophageal adenocarcinoma.

^a^Aspirin 9(15%), anti-platelet agent 4(6%), anticoagulants 1(2%).

**Table 2.** Cryoablation variables (62 patients, 174 CRYO sessions).

	Percentage (number)
Dysplasia ablated	100 (23)
IM ablated at >1 year follow-up endoscopy	97 (56/58)
At follow-up endoscopy^a^	100 (58/58)
BE recurrence – reablated in all	9 (5)
CRYOs per patient	Mean 2.8 range 1–14
CRYOs per cm BE	Mean 1.1 range 0.4–3
Minor adverse events (no major events) after CRYO session – percentage (number)	
Pain	4 (7)
Pain requiring only 1 dose acetaminophen	2 (3)
Odynophagia − 3 mild; 1 moderate^b^	2 (4)
Chest / epigastric pain − 3 mild; 1 moderate^b^	2 (4)
Fever (99–100°)	1 (1)
Blood (<4 ml) limiting CRYO dose by 25% 1 (1)	
Wheezing (likely sedation related) requiring albuterol nebulizer	1 (1)
Strictures	0 (0)

^a^Follow-up endoscopy in ≤12mo in two patients showed complete ­eradication of BE.

^b^Moderate is > 7 days.

All EAC or dysplasia was eradicated, including three patients with 10 to 14 cm very long HGD/IM (two white females, one black male) who otherwise were candidates only for esophagectomy. Nodular/superficial EAC was ablated in four who also had HGD in other areas. HGD in 7, and low grade dysplasia (LGD) and/or indefinite dysplasia (IDD) in 16 were ablated with CRYO. Four patients died in 16 months, 5 years, 9 years, and 10 years after their last CRYO from chronic aspiration (refusing fundoplication), chronic renal disease due to collagen vascular disease, a stroke, or intestinal ischemia, respectively.

BE recurred on endoscopic pathology in 9% (Table 2) in 53 ± 18mo from initial CRYO. BE recurred in 4/14 (29%) with initial LGD/IDD compared with 1/38 (3%) who initially had only non-dysplastic BE (relative risk 0.09 [95% CI,0.01, 0.76]; *p* < .03). CE was again accomplished for all recurrent IM using focal ablations with CRYO in five combined with APC in one for IM. Advanced disease recurred in only 1 female who had discontinued her PPI-BID because of insurance issues. Heartburn/reflux increased. Thirty months after CE of her BE with LGD, a polypoid adenoma with mucosal (T1a-LMP,-M2) carcinoma and HGD developed in a hiatal hernia, all re-ablated with multiple endoscopic methods without recurrence over 44 months. No patient initially with HGD or superficial EAC developed a recurrence.

For those with non-dysplastic BE, 5/38 (13%) developed small foci of IM in the cardia after CE. APC was used in 5/5 for CE of cardia IM. Focal areas of salmon-colored mucosa negative for IM were also treated prophylactically with APC in 15 patients combined with extra CRYO in 4. No second recurrence occurred either of BE over 33 months or of cardia IM over 20 months mean follow-up consistent with cure of BE in all patients.

Five patients had evidence of gastroparesis; of these, three developed IM in the cardia indicating an association (relative risk 0.03 [95% CI,0.09,0.96]; *p* < .04). Recurrence of IM after CE was associated with a significant hiatal hernia, initially >3cm (relative risk 0.08 [95% CI,0.01,0.65]; (*p* < .02), or increasing in size (relative risk 0.05 [95% CI,0.01,0.22]; *p* < .001). A hiatal hernia, present in most patients (Table 1), increased in size in 17%.

Seventy-nine percent of patients were not taking aspirin, anti-platelet medications or anticoagulants. No significant bleeding occurred after any CRYO session. No strictures occurred over an 80 months mean clinical follow-up. There were no emergency room visits. Only 1 patient required office follow-up sooner than the standard 10 days after CRYO to ensure medication compliance. Chest/epigastric pain or odynophagia were the most frequent adverse event (4%) after a CRYO session, lasting >1 week only once. Three patients took only one dose of acetaminophen. Non-circumferential application and/or dosimetry adjustments at subsequent sessions avoided repeat pain in those patients.

Compliance with medications for the 10 days after each CRYO session was 98%. Between sessions and after CE, 60% (twenty-nine) were compliant with at least PPI-BID [24] or dexlansoprozole daily (six) [6]. None of the thirty-five compliant patients [25] had recurrence of BE after CE. Of those that were not compliant (21%), seven were noncompliant [7] and five only partially compliant [5]. Four (33%) recurrences of BE were in those that were not compliant (relative risk 0.09 [95% CI,0.01,0.69]; *p* < .003). In 11 patients (19%) who decreased PPI to once daily, there was only 1 (9%) recurrence. When comparing patients that were compliant (PPI BID or daily) to those that were not compliant (Table 3), there were no significant differences in patient characteristics listed. No adverse effects from any dose of PPI occurred.

**Table 3.** Patient Variables for All, Compliant and Not compliant.

Patient variable*	All patients	Compliant^c^	Not Compliant
Signficant hiatal hernia^a^	26%	22%	29%
Nondysplastic BE	65.5%	65%	65%
Dysplasia	34.5%	35%	35%
Long segment BE	30%	22%	35%
Age > 59	46%	43%	48%
Race (white)	57%	57%	58%
Sex (female)	46%	48%	43%
Lymph nodes^b^	48%	67%	33%
Blood thinner use	22%	22%	23%

*Note:* BE: Barrett’s esophagus.

^a^Hiatal hernia initially >3 cm or increasing in size.

^ b^Lymph nodes on computerized tomography and/or endoscopic ultrasound. ^c^Includes patients taking PPI BID or daily.

*p Value = not significant for all variables.

On multivariant analysis noncompliance with high dose PPI (*p* < .001), a significant hiatal hernia (*p* ≤ .01), or gastroparesis (*p* ≤ .01) was associated with recurrence of either BE or cardia IM. Initial dysplasia was associated with recurrence of only BE (*p* = .03). Other variables were not associated with recurrence. Excluding the five patients with elements of gastroparesis, the recurrence rate of either BE or cardia IM was 35% (8/23) in those taking PPI only once daily or less compared with 0% (0/31) in those compliant with PPI-BID or dexlansoprozole daily (relative risk 0.04 [95% CI, 0.003, 0.73]; *p* < .001).

## Discussion

This study was designed to evaluate a method to eliminate IM/BE at any stage in a diverse population and assess over a long period of time the effectiveness and safety for prevention of recurrence and progression of BE. IM did not recur in any patients completing the course of CRYO including ablation of any recurrences; IM was ablated in 100% on endoscopic evaluation over a mean follow-up of more than 20 months. No study has reported this high a success rate over a more than 10 year follow-up. Only complex anti-reflux surgery designed to prevent any reflux of gastric secretions [26] has been shown to result in regression of Barrett’s columnar mucosa/IM to gastric mucosa in about 60% over about a four year follow-up. No patient with very long BE had regression after this surgery. Complete eradication was accomplished in three such patients with very long BE and HGD in this study. Any gastric mucosa seen in about 25% of our patients during the course of CRYOs resolved on PPI-BID or was ablated.

On an intention-to-treat analysis, all dysplasia was ablated in 100%; IM was ablated in 94% including the 4 of 62 patients stopping CRYO prior to CE. Patients with BE are highly likely to benefit from PPI-BID since there were no recurrences after CE with CRYO on this dose. Compliance to PPI-BID for effective reflux control reported by Komanduri et al. [12] was 63%, similar to our study. To support the use of BID-PPI, studies have shown a decreased rate of progression of BE to EAC/HGD by about 70% after two to three years in a dose response relationship to PPI [1,2,11]. Doubling PPI-BID dose is required to normalize acid to 100% and duodenogastroesophageal reflux to 80% in patients with long-segment BE [27].

The many issues in effective BE screening and surveillance have been well defined [1,2,7,8,13–16,28]. A decrease in the incidence of EAC with improved survival by treating HGD in BE is well documented [1,3–6,13–16]. However, patients with HGD comprise only a small part of patients at risk for EAC [5–8,17,18,29,30]. Also, a multicenter RFA study showed that 63% of patients progressing to EAC were found during ablation of IM with RFA prior to completing complete ablation [18].

A consensus article in 2015 from >50 participants from >50 institutions sets the stage for state-of-the-art management of BE worldwide [1]. There was at least 80% agreement of the Consensus Group that BE progresses to EAC in 0.5%/yr, supported by other recent guidelines [15,16]. However, in the United States about 9000 people get EAC/yr, most arising from BE [13,24,30]. The 140,000,000 population from 40 to 84yo (USA census bureau) is at most risk for BE. Two percent (about 2,800,000) will have BE [4,8,28]. Assuming a minimum of 8% with BE progress to dysplasia and 4% with dysplasia develop EAC per year, then the EAC incidence of 320/100,000/yr which equals the 9000 EAC reported/yr can be accounted for. Recent studies support the above calculations [28,29]. As discussed in several publications, treating any stage of BE before it becomes EAC should be as effective as removal of colon adenomas before they become CRC [8,31,32]. For comparison, a 50 yo white male has a risk of colorectal cancer of about 80/100,000/yr (260/100,000 for age >65). If any colon polyps are to be removed to decrease this risk, why would he not have BE ablated if present? Dysplasia in IM further increases the risk of developing EAC to a minimum of 2000/100,000/yr (2%). Further advantages of initial ablation over surveillance are that it would remove many concerns of missing dysplasia with random sampling during surveillance, low adherence to surveillance and interobserver variability of detection lesions or pathology diagnosis of dysplasia.

One of the most significant adverse effects of BE endoscopic ablation, esophageal strictures, did not occur in this study. The risk of stricture formation is 30% for photodynamic therapy, 4–6% for RFA and 11% for balloon cryotherapy [3–6,8,15,17,18,22,33]. It was only 3% for APC in the latest study which showed APC to be very effective long-term for treatment of BE with dysplasia [19]. CRYO ablation, however, has an even lower incidence of stenosis [20,21], likely ≤1–4%, none in this study.

Chest pain, the most common adverse event, occurs in 63% treated with APC [19]. With RFA, chest pain is reported to be much more frequent (>50%), severe and long-lasting than with CRYO. In the study of RFA [12] with both the lowest reported BE recurrence rate (4.8% down from 10.9% in their historical controls) and stricture rate (2.3%), pain was not discussed although acetaminophen with codeine was routinely prescribed. Acetaminophen was needed after a CRYO session in only 2% in this study which implies CRYO results in much less pain than RFA. At our institution acetaminophen/oxycodone was always given after RFA due to pain lasting one to two days in all patients and for about one week in approximately 15% after circumferential RFA. In most CRYO studies at least 15% have pain compared with only 5% pain rate in this current study, most likely because of high doses of medications given to limit injury from reflux [23] and the different dosimetry and pattern of delivery of the liquid nitrogen. In our study APC in the esophagus has been quite useful without any side-effects for ablation of minor focal lesions and for IM in the cardia. In another recent study [20], pain at 48 h after ablation occurred in 29% after CRYO with liquid nitrogen administered for two 20 s long cycles per area ablated compared with 73% pain in those that received circumferential RFA. This 73% pain rate is substantially higher than the mean 3.3% noted in a meta-analysis of RFA [6]. But meta-analyses are limited in that exclusion criteria act as measurements applied to the study population [34] and as such, exclusion of even one study or trial can influence the results and the study’s general applicability [35].

Other significant adverse effects with RFA or APC such as fever in up to 11%, mediastinitis (<1%), bleeding (1–3%), perforations (1–2%), effusions (<1%), or pain requiring hospital admission have been reported [6,12,15–20], all probably due to potential focal injury of deeper layers of the uneven esophageal wall. CRYO causes the desired necrosis of only the epithelial and superficial submucosal layers in all patients with mild resolving inflammation of the muscularis propria in only 40% at doses of liquid nitrogen for fewer than 20 s [25].

Statistically significant risk factors for recurrence of BE in this study were: (1) not taking PPI-BID; Dexlansoprazole daily may be equivalent; (2) a significant hiatal hernia; (3) gastroparesis; and (4) initial LGD or IDD. Some of these risk factors for recurrence have also been noted after RFA [6,12,13,17]. These factors are all associated with increased exposure of the esophagus to acid, bile and other gastric secretions which cause BE [13,26].

Risk factors for EAC have been defined [1–8]. Nevertheless, the likelihood of finding BE in lower risk subgroups is still about 40% of the risk in the highest risk subgroup, white males that are age >50yo with large abdominal girth and reflux symptoms [14,28]. This study also found that EAC with BE can occur in patients that are female, black, or without reflux symptoms. While this series achieved high statistical significance over a long mean follow-up, it also found the occurrence of relatively infrequent events after CE of BE such as a metachronous adenocarcinoma in the cardia. Three other similar cases have been reported after RFA for BE [36].

Even when lower than the usual values of 0.32% for nondysplastic BE progressing to EAC and 4% for dysplasia progressing to EAC are used for calculations, the number-needed-to-treat for this cohort is 51, which supports the number-needed-to-treat calculations of 23 to 45 by Ganz [37]. Recently, two studies further support endorsement of a more consistent and effective approach to management of all stages of BE. Frei et al. [38] validated a tissue systems pathology assay for BE that identified a significant subset of nondysplastic BE patients who progress at a rate comparable with published estimates for expert-confirmed low-grade dysplasia, identifying most future progressors to HGD and/or EAC within five years. Krishnamoorthi et al. [39] reported that the incidence of HGD and/or EAC is similar for BE-IDD and BE-LGD. They recommended the same surveillance and treatment.

Limitations of this study are that it is from one endoscopist, and there is no randomized control group receiving no treatment. This, however, was by design since in 2008 there was no cohort study of CRYO to define treatment variables including high-dose PPI and other anti-reflux treatment during CRYO, nor its long-term safety and efficacy. At the time initial RFA data were being published so a no treatment control group or a ramdomized controle trial with other ablation methods was not possible. In addition there is at most only minimal complete regression of BE with PPI treatment; only slower progression to dysplasia has been shown.

My experience with CRYO since its early use allowed interim refinements to the CRYO method showing its safety and efficacy over >10 years. At the time the study was designed in 2008 TruFreeze CSA, Inc. initially recommended 10 s of freeze four times per site which has changed over the years based on collective investigators experiences without any comparative studies. They now recommend 20 s liquid nitrogen for two times per site as reasonable. At our center 15 s for 3 times per site is now the usual preferred dose. There are no guidelines or recommendations for continuous high-dose PPI use or other anti-reflux measures for treatment of BE. This study shows how high dose PPI can be of benefit.

The effectiveness of RFA including cost-effectiveness for non-dysplastic BE has been demonstrated [8,12,15–18,20]. Statistical models show that endoscopic ablation of BE without any further surveillance is cost-effective (willingness-to-pay = $50,000/quality-adjusted-life-year) even if nondysplastic BE/IM is successfully ablated in only 47% of patients [5,8,16]. Conservative theoretical cost calculations (Table 4) for the management of nondysplastic BE (≥C1:M2) indicates that initial CRYO is cost-effective compared with standard surveillance followed by ablation/treatment only as needed if BE disease progresses. For typical management over 16 years, a potential cost savings of 9% ($1109 cost per patient) would occur when comparing the initial surveillance Group1 ($13,545 cost per patient) with the initial treatment Group2 ($12,436 cost per patient) for follow-up and treatments as outlined using payments generated from the perspective of the payer based on standard government reimbursement in our region. APC may be even more cost effective [19].

**Table 4.** Nondysplastic Barrett’s esophagus management costs comparing initial surveilance versus initial treatment over 16 years.

	Initial treatment	Endoscopy surveillance	Pathology sites	Further treatment	Costs^a^ per patient
Group #1					
(Standard surveillance; treatment for dysplasia when it occurs)	None	Six times: once at one year then every three years (total = 5)	100% of patients (typical = three containers with some special stains). WATS only twice.	Only as needed in 10% (assuming 10% develop dysplasia treated with ablation at least twice)	$13,545
Group #2					
(CRYO treatment; then only limited surveillance)	One CRYO at 6–12 months with pathology	Every five years (total = 3)	34% − three typical containers WATS – none	33% – Second CRYO followed by ≤ 3 endoscopies q5 year (assume 2% develop recurrence or dysplasia treated with no more than two more ablations)	$12,436

*Notes:* Group #1 (standard surveillance with treatment for dysplasia only when it occurs): Six surveillance endoscopy at one year then every three years with typical pathology (three containers with some special stains) and wide-area transepithelial sampling (WATS) only twice [7,40] shown to be potentially effective. Then, assume that only 10% will develop dysplasia that needs endoscopic ablation at least twice (likely more). Group #2 (CRYO treatment with limited surveillance as below): One CRYO (at 6–12 months with pathology). Then, three surveillance endoscopies every five years with typical pathology (no WATS) in one-third. In another third assume the need for a second CRYO followed by ≤3 endoscopies every five years. For the final third assume the need for another extra CRYO followed by two endoscopies every five years. Finally, assume that ≤2% will develop recurrence or dysplasia that needs no more than two ablations.

^a^Cost/patient for Group #1 is $1109 more compared with Group #2 over first 16 years. If over the next 15 years standard surveillance continues in Group #1 but not in Group #2 except for those who did recur, the management for Group #2 generates about 50% less cost over 30 years. Costs include hospital/facility, global pathology, endoscopic accessories, WATS sampling kit, truFreeze ablation kit and payments to the endoscopist, pathologist and anesthesiologist.

Despite the above, and the lack of consensus [1] as to how and when to perform surveillance and treatment of BE, surveillance at three to five years is still the accepted guideline even though the ‘quality of evidence is very low and the strength of recommendation is conditional’ [15]. That the progression of NDBE to advanced EAC in less than three years is possible is well known [41]. The diagnosis of even NDBE frequently causes patient anxiety, misunderstanding and other health-related quality of life issues [13,42]. Informed consent for treatment of NDBE should include treatment as an option. The incidence of EAC should be greatly diminished if both the stimulus that causes BE and the presence of goblet cells are treated [43,44] with maximum PPI, diet changes and ablation of IM in a manner that maximizes cost-effectiveness and safety. The message of the editorial, ‘Enough evidence, time to act’ [45], regarding treatment of low-density lipoprotein cholesterol, may now equally apply to endoscopic ablation treatment of any patient with BE [8,28,31,32]. In conclusion, a multicenter outcomes trial for any stage of BE comparing PPI-BID with standard surveillance to PPI-BID with initial CRYO, APC or RFA and only limited surveillance would further define cost-effective treatment of BE.

## Supplementary Material

Supplemental Material

## Data Availability

The author confirms that the data supporting the findings of this study are available within the article [and/or] its supplementary materials. Data are available for review.
