# Correction

**DOI:** 10.1080/07853890.2023.2242207

**Published:** 2023-08-08

**Article title:** Improving clinical outcomes of Barrett’s esophagus with high dose proton pump inhibitors and cryoablation

**Authors:** Snady, H.

**Journal:** *Annals of Medicine*

**Bibliometrics:** Volume 55, Number 01, pages 1256–1264

**DOI:** https://dx.doi.org/10.1080/07853890.2023.2191002

The article was processed and published with references 24 to 35 incorrectly ordered. The references have now been renumbered as per intended making sure that the numerical order of citations in the text is retained as such. The article also has one minor layout correction each in Tables 1, 2 and 4.

